# Conserved Molecular Players Involved in Human Nose Morphogenesis Underlie Evolution of the Exaggerated Snout Phenotype in Cichlids

**DOI:** 10.1093/gbe/evad045

**Published:** 2023-03-17

**Authors:** Anna Duenser, Pooja Singh, Laurène Alicia Lecaudey, Christian Sturmbauer, R Craig Albertson, Wolfgang Gessl, Ehsan Pashay Ahi

**Affiliations:** Institute of Biology, University of Graz, Graz, Austria; Institute of Biology, University of Graz, Graz, Austria; Aquatic Ecology Division, Institute of Ecology and Evolution, University of Bern, Bern, Switzerland; Swiss Federal Institute of Aquatic Science and Technology (EAWAG), Kastanienbaum, Switzerland; Institute of Biology, University of Graz, Graz, Austria; Department of Natural History, NTNU University Museum, Norwegian University of Science and Technology, Trondheim, Norway; SINTEF Ocean, Aquaculture Department, Trondheim, Trøndelag, Norway; Institute of Biology, University of Graz, Graz, Austria; Department of Biology, University of Massachusetts, Amherst, Massachusetts, USA; Institute of Biology, University of Graz, Graz, Austria; Institute of Biology, University of Graz, Graz, Austria; Organismal and Evolutionary Biology Research Programme, University of Helsinki, Helsinki, Finland

**Keywords:** RNA-seq, Lake Malawi, Lake Tanganyika, snout flap, cichlids, functional conservation

## Abstract

Instances of repeated evolution of novel phenotypes can shed light on the conserved molecular mechanisms underlying morphological diversity. A rare example of an exaggerated soft tissue phenotype is the formation of a snout flap in fishes. This tissue flap develops from the upper lip and has evolved in one cichlid genus from Lake Malawi and one genus from Lake Tanganyika. To investigate the molecular basis of snout flap convergence, we used mRNA sequencing to compare two species with snout flap to their close relatives without snout flaps from each lake. Our analysis identified 201 genes that were repeatedly differentially expressed between species with and without snout flap in both lakes, suggesting shared pathways, even though the flaps serve different functions. Shared expressed genes are involved in proline and hydroxyproline metabolism, which have been linked to human skin and facial deformities. Additionally, we found enrichment for transcription factor binding sites at upstream regulatory sequences of differentially expressed genes. Among the enriched transcription factors were members of the FOX transcription factor family, especially *foxf1* and *foxa2*, which showed an increased expression in the flapped snout. Both of these factors are linked to nose morphogenesis in mammals. We also found *ap4* (tfap4), a transcription factor showing reduced expression in the flapped snout with an unknown role in craniofacial soft tissue development. As genes involved in cichlid snout flap development are associated with human midline facial dysmorphologies, our findings hint at the conservation of genes involved in midline patterning across distant evolutionary lineages of vertebrates, although further functional studies are required to confirm this.

**Significance**

The study of the evolution of similar physical traits across taxa can give insight into the molecular architecture underlying shared phenotypes. This has mostly been studied in bony structures, whereas soft tissue traits have been less intensely covered. We investigated the exaggerated snout in cichlid species from Lake Malawi and Lake Tanganyika and found that many genes involved in the development of the snout flap and are also associated with midline dysmorphologies in humans, implying a conservation across distant vertebrate lineages.

## Introduction

The repeated evolution of phenotypes, reflecting particular ecological specializations, is a ubiquitous characteristic of adaptive radiations (Schluter and Nagel 1995; Losos et al. 1998; Rüber et al. 1999; Rundle et al. 2000). Cichlid adaptive radiations from the East African Great Lakes display an impressive array of repeated morphological traits (Kocher et al. 1993), including a few dramatic examples of exaggerated phenotypes like the overgrowth of craniofacial soft tissues in various anatomical regions such as lips (Colombo et al. 2013; Manousaki et al. 2013; Machado-Schiaffino et al. 2014; Baumgarten et al. 2015; Henning et al. 2017; Lecaudey et al. 2019), the frontal head (nuchal hump) (Lecaudey et al. 2021), and the nose snout (or nose flap) (Concannon and Albertson 2015; Conith et al. 2018). Although there is increasing insight into the evolution of such phenotypic novelties, especially hypertrophid lips, exaggerated soft tissue traits are less well studied than bony traits and the genetic mechanisms underlying these traits are not entirely understood. Comparative approaches can shed light on the genetic mechanisms that reconfigure the body plan and give rise to such complex traits. With examples of both parallel and non-parallel mechanisms underlying cases of repeated evolution (e.g., Colombo et al. 2013; Manousaki et al. 2013) of phenotypic novelties, such comparisons can thus help us to understand the molecular mechanisms that shape morphological diversity.

**Fig. 1.**
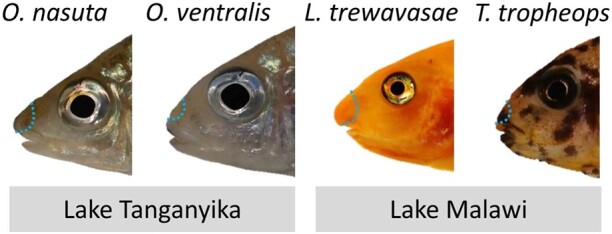
Convergent cases of snout flap evolution. East African cichlid species used in this study. The area of the soft tissue that was dissected is depicted by dashed lines. *O. nasuta*, *Ophthalmotilapia nasuta*; *O. ventralis*, *Ophthalmotilapia ventralis*; *L. trewavasae*, *Labeotropheus trewavasae*; *T. tropheops*, *Tropheops tropheops*.

One such exaggerated repeated phenotype in cichlids is the snout flap, a pronounced projection that emanates from a flap of fibrous tissue just above the upper lip. It is a rare morphological innovation that has only evolved in two tribes of cichlid fishes from East Africa, the modern haplochromines in Lake Malawi and the Ectodini in Lake Tanganyika (fig. 1) (Concannon and Albertson 2015). When this snout is sexually monomorphic, it is thought to be a trophic adaptation that improves feeding efficiency (Konings 2007). When the snout is sexually dimorphic, it is hypothesized to be involved in sexual selection (Konings 2007; Concannon and Albertson 2015). The cichlid snout flap has been studied at the molecular level only in the genus *Labeotropheus* from Lake Malawi where it is sexually monomorphic and functions as a trophic adaptation to efficiently leverage algae from rocks (Concannon and Albertson 2015; Conith et al. 2018). A similar snout structure has also been described in two species from the Ectodini tribe (*Ophthalmotilapia nasuta* and *Asprotilapia leptura*) from Lake Tanganyika. In *A. leptura*, it is sexually monomorphic and likely involved in increased foraging efficiency (similar to *Labeotropheus*), whereas in *O. nasuta*, it is only found in mature males and is likely a secondary sexual character (Hanssens et al. 1999; Conith et al. 2019). Thus, the exaggerated snout is a convergent phenotype that evolved independently in two cichlid lineages that diverged >9.0 Ma (Irisarri et al. 2018; Conith et al. 2019).

In *Labeotropheus*, the snout is evident histologically by the time the yolk is absorbed and exogenous feeding occurs (∼1 month postfertilization) (Concannon and Albertson 2015; Conith et al. 2018), and the early formation and growth of the snout are linked to the transforming growth factor beta (TGFβ) signaling pathway (Conith et al. 2018). However, it remains unclear which 1) genes and pathways contribute to the maintenance of this complex trait (i.e., its genetic architecture) and if 2) these candidate genes and pathways can be linked to more conserved patterning of craniofacial features. Furthermore, whereas previous research focused on the TGFβ signaling pathway, a more extensive molecular interaction map of the formation and maintenance of this exaggerated phenotype remains to be unraveled. A transcriptome-wide overview is particularly important because it is well-known that there is molecular crosstalk between the TGFβ signaling pathway and several other pathways which all play a pivotal role in craniofacial morphogenesis and adaptive evolutionary divergence in teleost fishes (Ahi 2016).

In this study, we set out to investigate the molecular mechanisms that underlie the formation and evolution of the exaggerated snout phenotype, in two non-sister cichlid lineages from Lakes Tanganyika and Malawi (fig. 1), and link it to conserved molecular players in midline patterning in other vertebrates. We compared two species that develop a snout: 1) *Labeotropheus trewavasae* (tribe Haplochromini) from Lake Malawi and 2) *O. nasuta* (tribe Ectodini) from Lake Tanganyika (fig. 1). As controls, we used two closely related species within the same tribes that do not develop such a structure: 1) the Lake Malawi mbuna species *Tropheops tropheops* (Haplochromini) and 2) the Lake Tanganyika featherfin cichlid *Ophthalmotilapia ventralis* (Ectodini) (fig. 1). We used mRNA-sequencing to quantify gene expression differences between the exaggerated snout and non-snout tissues for each lake. Altogether, we identified parallel and non-parallel molecular mechanisms that underlie the evolution of the snout flap in Lake Malawi and Lake Tanganyika cichlids. Our study design provides valuable information into conserved and unconserved regulatory mechanisms underlying the morphogenesis of a unique hypertrophic facial soft tissue in cichlids, which also exhibit striking similarity to those mechanisms driving craniofacial development and midline patterning in other vertebrates including humans. Cichlids have been proposed as an excellent model system to study craniofacial skeletal deformities in humans (Powder and Albertson 2016), and our study is one of the first indications that cichlids can be used as models to study deformities in facial soft tissues as well.

## Results

To investigate molecular mechanisms underlying the formation of a snout flap in two distant lineages of cichlids, we dissected the snout tissue of five biological replicates per species, which entailed the area above the upper lip including the nostrils. These tissue samples consisted of epidermis, dermis, and the underlying connective tissue (fig. 1). Subsequently, we extracted RNA of these five samples per species to quantify gene expression differences.

### RNA-Sequencing, Gene Expression, and Downstream Analyses

The RNA-sequencing resulted in between 6.7 and 15.8 million reads per sample, and after filtering of low-quality reads, between 4.6 and 11.1 million reads were retained for each sample (supplementary table S1, Supplementary Material online). The raw data of sequence reads have been deposited in the Sequencing Read Archive (SRA; supplementary table S1, Supplementary Material online) of NCBI (accession number: PRJNA770252). The final annotation of all merged species included 33,251 genes. Through pairwise comparisons between species of each lake radiation, we identified 832 of the 33,251 genes (2.4%) with significant differential expression (false discovery rate (FDR) cutoff at *P* < 0.05) for the comparison of *O. nasuta* versus *O. ventralis*, while the comparison between *L. trewavasae* and *T. tropheops* yielded 4,292 (12.7%) significant differentially expressed genes (FDR cutoff at *P* < 0.05).

Gene ontology (GO) enrichment analysis conducted for differentially expressed genes within each species pair comparison for Lake Tanganyika and Lake Malawi, respectively, revealed the involvement in biological processes like “peptidyl-proline modification,” “tendon development,” and “cell adhesion” for the comparison of the Lake Tanganyika species (*O. nasuta* vs. *O. ventralis*), while the Lake Malawi comparison (*L. trewavasae* vs. *T. tropheops*) showed terms like “cell matrix adhesion,” “apoptotic process involved in morphogenesis,” as well as “regulation of brown fat cell differentiation” among more cell-specific processes (supplementary table S2, Supplementary Material online).

**Fig. 2.**
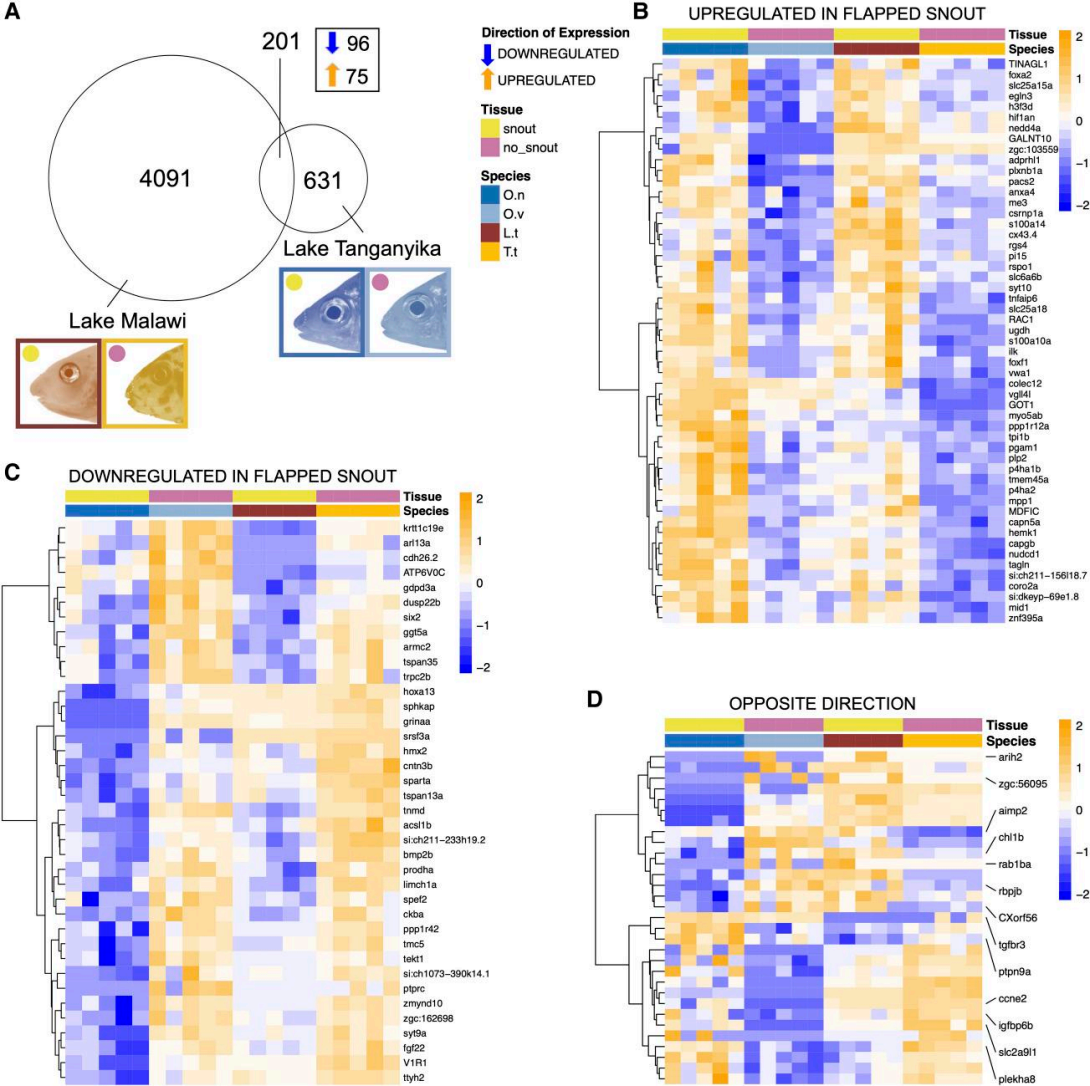
Differentially expressed genes in the snout regions. (*A*) Venn diagram of genes with differential expression between the snout regions (“snout” and “no snout”) for Lake Malawi and Lake Tanganyika and the overlap of 201 genes between the two comparisons of which 96 are downregulated and 75 are upregulated in the flapped snout of both comparisons. Dendrogram clusters of the overlapping annotated genes showing upregulation (*B*) and downregulation (*C*) in expression in the flapped snout tissue, as well as those showing differential expression in both comparisons but in opposing directions (including not annotated genes) (*D*). Orange and blue shadings indicate higher and lower relative expression, respectively. Lake Tanganyika: *Ophthalmotilapia nasuta* (O.n, dark blue), *Ophthalmotilapia ventralis* (O.v; light blue); Lake Malawi: *Labeotropheus trewavasae* (L.t; red), *Tropheops tropheops* (T.t; orange).

To understand if similar genes were involved in the formation of a snout across the two lakes, we investigated the intersection set of the two pairwise comparisons and could identify an overlapping list of 201 differentially expressed (DE) genes which were distinct between the flapped snout versus the non-flapped snout regions in both lakes (24.2% of the differentially expressed genes [DEG] for the Lake Tanganyika comparison and 4.8% for the Lake Malawi comparison) (fig. 2*A*) (supplementary table S3, Supplementary Material online). Among the shared DE genes, 84.6% showed the same direction of expression with 74 genes being upregulated and 96 genes being downregulated in the flapped snout tissues in both comparisons, which is a higher number of shared expression direction than one would expect by chance (hypergeometric test, *P* < 0.05), whereas 31 genes showed expression differences in opposite directions across the comparisons for each lake (fig. 2*B*–*D*). The heat map clustering of the DE genes showed that there are at least two major branches in each group of up- or downregulated gene sets, while the clustering of the DE genes with opposite expression pattern also revealed the presence of two major branches (fig. 2*B*–*D*). These clustering structures indicate distinct transcriptional regulations within each group which potentially originated from the effects of different upstream regulators.

We performed GO enrichment analysis using the list of the shared 201 DE genes as the input, and the result showed significant enrichment of GO terms for several biological processes such as amino acid metabolism (particularly proline-related metabolic processes), “tendon development,” “positive regulation of BMP signaling pathway,” and cell adhesion and cell fate (supplementary table S2, Supplementary Material online). When dividing the genes in their direction of expression in the snout flap, GO enrichment for upregulated genes was associated with “peptidyl-proline hydroxylation,” “tendon development,” “muscle attachment,” “endothelial cell development,” “negative regulation of Notch signaling pathway,” and, although not significantly, “positive regulation of Wnt signaling pathway.” The downregulated genes were involved in a lot of terms related to cell fate commitment and negative regulation of cell fate as well as “proline catabolic process” and “positive regulation of BMP signaling pathway” (supplementary table S2, Supplementary Material online).

**Fig. 3.**
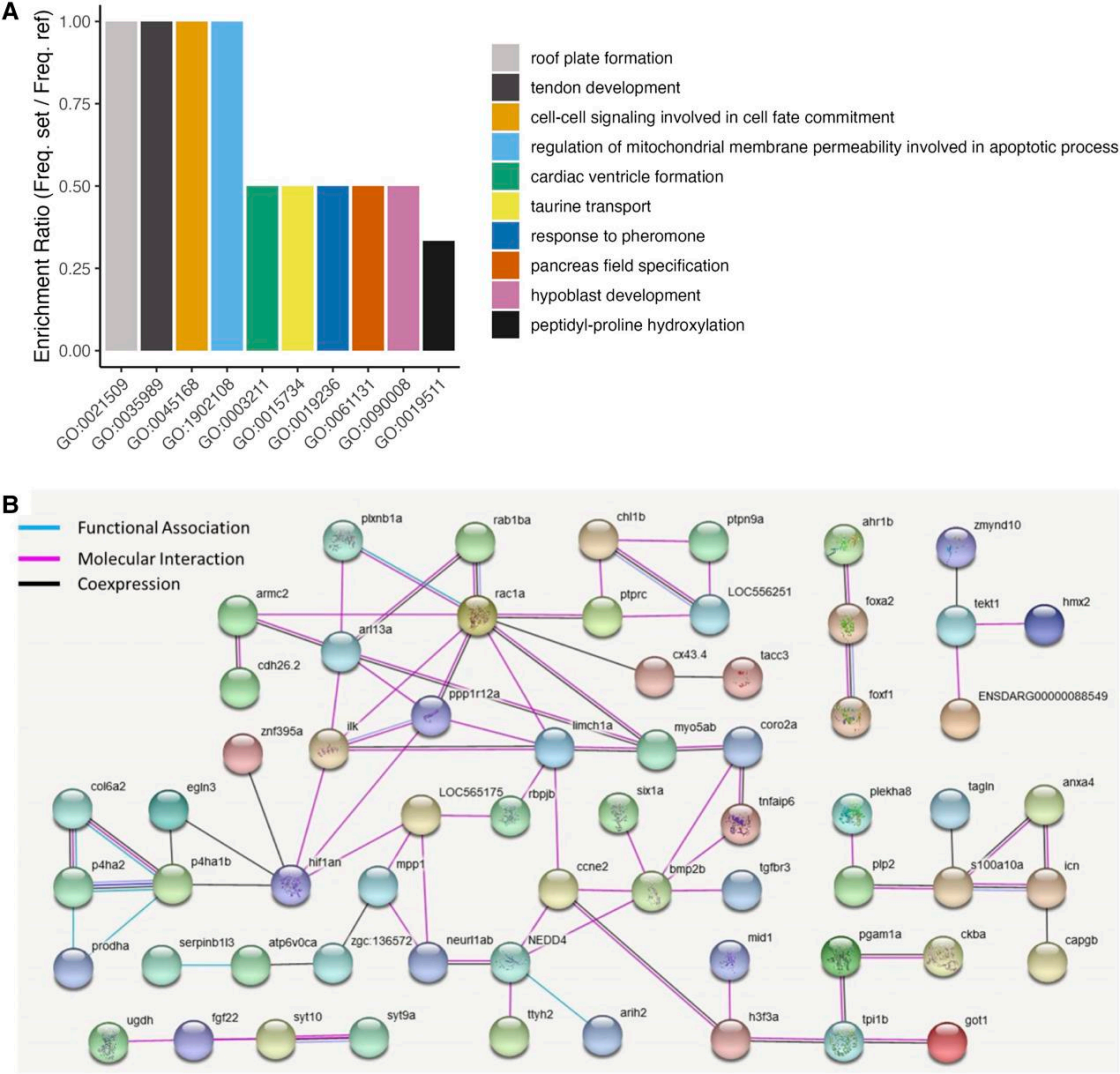
Functional analyses of the overlapping differentially expressed genes in the flapped snout. (*A*) Enrichment for gene ontologies of biological processes using the shared 201 differentially expressed genes. (*B*) Functional interactions between the differentially expressed genes predicted based on zebrafish databases in STRING v10 (http://string-db.org/).

We also applied the same list of the shared 201 DE genes for interactome analysis which demonstrated a large, interconnected network of genes with molecular and functional associations. Some of the genes in the network formed an interaction hub with the highest level of associations (based on the number of predicted interactions with other DE genes) with other genes such as *bmp2b*, *hif1an*, and *rac1a*, suggesting their more pivotal role in the formation of the flapped snout structure in cichlids (fig. 3*B*). Furthermore, we conducted transcription factor (TF) binding motif overrepresentation analysis on the upstream regulatory sequences of the DE genes through MEME tool (Bailey et al. 2009). In total, seven motifs were enriched on the upstream regulatory sequences of at least 40 out of 201 DE genes (table 1). Next, we checked the similarities of the enriched motifs with known TF binding sites in vertebrates, and at least 11 TF candidates were identified to potentially bind to those motifs.

**Table 1:** Predicted Motifs and Upstream Regulators Potentially Binding to Them

TF Binding Site	PWM ID	Count	Predicted Motif Sequence	*E*-Value
FOXP1	M00987	71/201	AMAMACAMAMAMAMAMACACACAMAMACA	3.85E−12
FOXJ1	M00742			3.52E−08
RREB1	MA0073.1			1.87E−07
FOXJ1	M00742	47/201	AAAAASAAAMAAAMWMWCWKT	8.69E−10
FOX	M00809			9.15E−07
FOXD3	MA0041.1			3.95E−07
SP1	MA0079.2	41/201	CHCCYCCYCCYCCSCYCTCCY	1.12E−08
IRF9	M00258	61/201	KTTTTTYTTTYYCWK	2.90E−09
MEF2	M00405	72/201	RTTAAAAAAAA	4.28E−08
AP4	M00927	93/201	CWGCTGCWGCTKSTS	7.38E−08
HEB/tcf12	M00698	66/201	NYYCTGCTGD	1.03E−06

Note.—Enriched motifs on upstream regulatory sequences of the DE genes are presented in degenerated sequence format. PWD IDs indicate positional weight matrix ID of predicted binding sites, and *E*-values refer to matching similarity between the predicted motif sequences and the PWD IDs. The count implies the number of genes containing the predicted motif sequence on their regulatory region.

### Expression Analysis by qPCR

Validation of DE genes from RNA-seq was accomplished via real-time quantitative polymerase chain reaction (qPCR), normalized to stably expressed reference genes (Kubista et al. 2006). In our previous studies of East African cichlids, we found that validation of reference gene(s) is an essential step as genes only selected from literature are not necessarily the best choice and can vary a lot between different species and tissue types (Ahi, Singh, et al. 2019; Ahi, Richter, et al. 2019; Pashay Ahi and Sefc 2018; Ahi et al. 2020a, 2020b). We chose six candidate reference genes with a small log_2_ fold change and the lowest coefficient of variation (CV) throughout all the samples (supplementary table S4, Supplementary Material online). Based on the rankings by the three software tools, BestKeeper, geNorm, and NormFinder, only one of the candidate reference genes, *pak2b*, showed consistent stability, that is, always ranked among top two most stable reference genes (table 2). Thus, we used the Cq value of *pak2b* in each sample to normalize the relative gene expression levels of our target genes.

**Table 2:** Ranking of Reference Genes in the Nose Tissue Samples Using Three Different Algorithms

BestKeeper				geNorm		NormFinder	
Ranking	SD	Ranking	*r*	Ranking	*M*	Ranking	SV
*sp3*	0.461	*pak2b*	0.94	*pak2b*	0.369	*nup58*	0.310
*pak2b*	0.471	*pphln1*	0.931	*flot2a*	0.386	*pak2b*	0.408
*flot2a*	0.491	*flot2a*	0.926	*pphln1*	0.397	*pphln1*	0.443
*nup58*	0.509	*vps26a*	0.916	*sp3*	0.418	*flot2a*	0.498
*vps26a*	0.551	*nup58*	0.889	*nup58*	0.427	*sp3*	0.518
*pphln1*	0.587	*sp3*	0.887	*vps26a*	0.428	*vps26a*	0.646

SD, standard deviation; *r*, Pearson product–moment correlation coefficient; SV, stability value; *M*, *M*-value of stability.

**Fig. 4.**
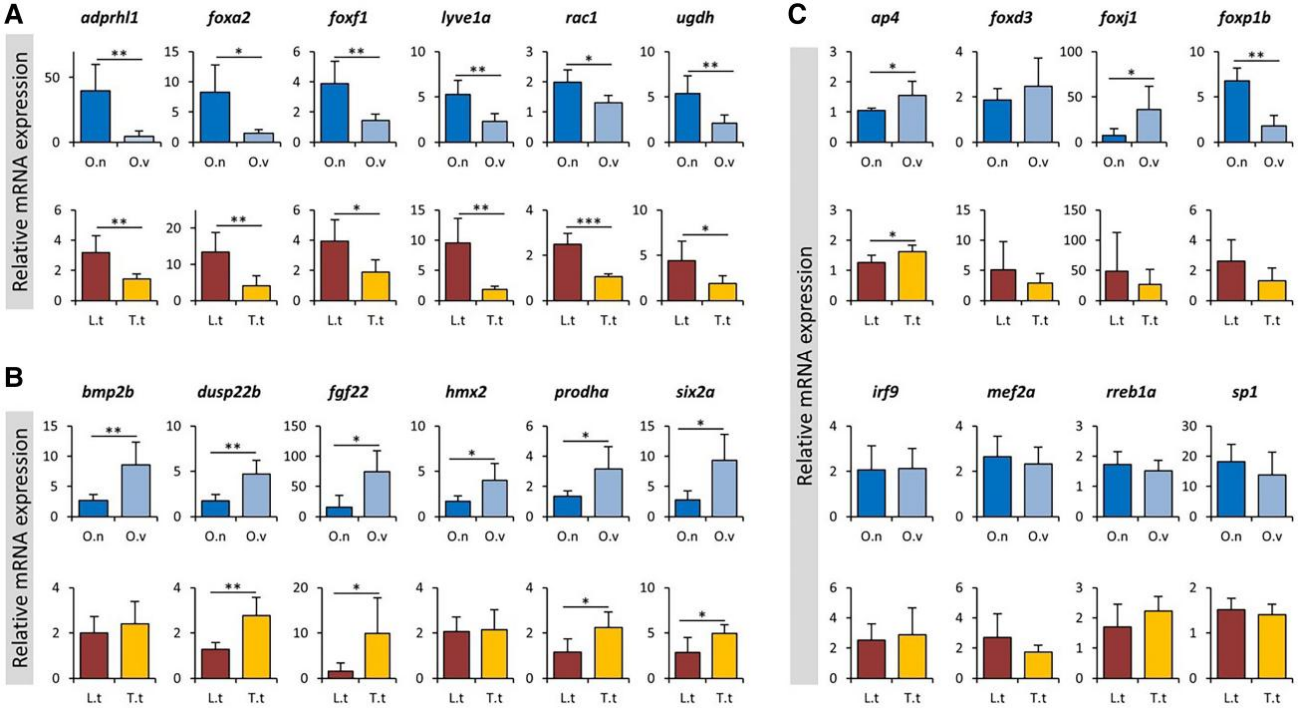
qPCR expression analysis of a selected set of candidate genes. qPCR validation of expression differences for selected sets of genes showing upregulation (*A*) or downregulation (*B*) in snout tissues. (*C*) qPCR expression analysis of predicted transcription factors. The bars indicate mean and standard deviation of RQ expression values for five biological replicates per species. The asterisks above the bar represent significant expression differences (*P < 0.05; **P < 0.01; and ***P < 0.001). O.n, *Ophthalmotilapia nasuta* (dark blue); O.v, *Ophthalmotilapia ventralis* (light blue); L.t, *Labeotropheus trewavasae* (red); T.t, *Tropheops tropheops* (orange).

Among the DE genes identified by RNA-seq, we chose 12 genes with a known role in nose morphogenesis and/or other related functions in craniofacial development mainly based on genetic studies in humans (table 3), together with eight predicted upstream TFs (including *ap4*, *foxd3*, *foxj1*, *foxp1*, *irf9*, *mef2a*, *rreb1a*, and *sp1*) for qPCR analysis (fig. 4).

**Table 3:** A Selected Set of Differentially Expressed Genes in the Flapped Snout Regions of Studied Cichlids With Known Related Functions in Nose Morphogenesis in Mammalian Models

Gene	Related Function	Organism	References
*adprhl1*	Duplication of this gene is associated with prominent forehead, short and bulbous nose, and broad philtrum	Human	(De Pater et al. 2005)
*bmp2*	A ligand of the TGFβ signaling and its monoallelic deletion is associated with short upturned nose and long philtrum	Human	(Tan et al. 2017)
*dusp22*	Deletion at terminal end of this gene is associated with saddle shape nose morphogenesis	Human	(Hosono et al. 2020)
*fgf22*	Genomic rearrangement encompassing this gene is associated with elongation of nose with prominent nasal bridge	Human	(Quigley et al. 2004)
*foxa2*	Both deletion and missense variation in this gene cause hallow nasal bridge, short upturned nose, and downturned nasolabial folds	Human	(Dines et al. 2019)
*foxf1*	Duplication and triplication cause bulbous nose and wide nasal bridge	Human	(Kucharczyk et al. 2014)
*hmx2*	Hemizygous deletion in this gene causes broad nasal bridge and prominent nose	Human	(Miller et al. 2009)
*lyve1*	Dysregulation of this gene is associated with cutaneous angiosarcoma on the nose	Human	(Mitteldorf et al. 2018)
*prodh*	Deletion and/or missense mutations in this gene cause frontal bossing, thin upper lip, and short nose	Human	(Guilmatre et al. 2010)
*rac1*	Loss-of-function mutation in this gene causes failure in fusion of medial nasal processes and prominent nasal bridge	Human, mouse	(Thomas et al. 2010; Reijnders et al. 2017)
*six2*	Deletion in this gene causes frontonasal dysplasia syndrome in human with nasal clefting and broad nasal tip, and developmental deformities in nasal bridge in mouse	Human, mouse	(Hufnagel et al. 2016; Okello et al. 2017)
*ugdh*	Missense mutation in this gene causes bulbous nose and smooth philtrum	Human	(Alhamoudi et al. 2020)

Based on the RNA-seq results, six of these candidate genes displayed upregulation in expression in the flapped snout (*adprhl1*, *foxa2*, *foxf1*, *lyve1a*, *rac1*, and *ugdh*), while the six other candidate genes (*bmp2b*, *dusp22b*, *fgf22*, *hmx2*, *prodha*, and *six2a*) showed a downregulation in expression in the flapped snout. The results of qPCR analysis confirmed that almost all of the genes showed expression patterns similar to RNA-seq results, except for *bmp2b* and *hmx2* which showed no significant difference between the snout regions of *L. trewavasae* and *T. tropheops*. Among the predicted TFs, only *ap4* showed consistent differences across both comparisons displaying a slightly reduced expression in both species with protruded snouts (*O. nasuta* and *L. trewavasae*). This indicates potential transcriptional repressor effects of *ap4* on the downstream genes in the hypertrophic snout region. Two predicted members of FOX transcription factors, *foxj1* and *foxp1*, showed expression differences but only in one of the comparisons (*O. nasuta* vs. *O. ventralis*), which makes them unlikely candidates for upstream regulators of shared DEGs in both comparisons. Altogether, the qPCR results demonstrate consistency between RNA-seq and qPCR results confirming the validity of our transcriptome data analysis.

## Discussion

Cases of repeated morphological evolution can contribute significantly to our understanding of the molecular architecture underlying repeated phenotypes. The snout flap of the Lake Malawi cichlid *L. trewavasae* is thought to have evolved through natural selection (Concannon and Albertson 2015) as it plays a distinct role in the foraging efficiency for algal scraping (Konings 2007; Conith et al. 2019). No difference in snout flap size has been detected between male and female of *Labeotropheus*, and its formation has been shown to coincide with the developmental time point when independent foraging begins, further supporting its ecological function (Concannon and Albertson 2015). In contrast, in Lake Tanganyika, only *O. nasuta* males show distinct snout flaps, implying a role in mate choice that evolved through sexual selction (Concannon and Albertson 2015). Both sexes of *O. nasuta* are planktivorous suction feeders, a feeding adaptation that is presumably not enhanced by a snout flap, although the snout of males continues to grow with increasing age (Hanssens et al. 1999). The morphological similarity of the snout flap across two cichlid radiations allows us to investigate if conserved molecular players are involved in the formation of a snout, even if the morphologies possess different functions and differ in tissue composition and life history.

We found differing numbers of DE genes between the comparisons within Lake Malawi and Lake Tanganyika, with roughly five times more differentially expressed genes between the chosen species pair from Lake Malawi over the species pair from Lake Tanganyika. This, most likely, cannot be explained by the use of differing genera for the Lake Malawi comparison as the species flock shows a low sequence divergence of 0.1–0.25% probably due to the young age of the radiation (Malinsky et al. 2018) (supplementary fig. S1, Supplementary Material online), but could be an indicator for a difference in tissue composition. Comparisons of snout flap tissues have found that the snout of *Labeotropheus fuelleborni* contains a lot more intermaxillary ligament and loose connective tissue (80%) than the snout of *O. nasuta* (50%) (Conith et al. 2019). Additionally, the GO enrichment analyses for the two within-lake comparisons showed quite distinct enrichment terms. GO enrichment analysis for DE genes within the Lake Malawi comparison between *L. trewavasae* and *T. tropheops* yielded terms like “cell–matrix adhesion,” “regulation of brown fat cell differentiation,” and “apoptotic process involved in morphogenesis” among terms involved in nerve development and different terms not readily connected to snout morphology (supplementary table S2, Supplementary Material online). This could hint at a stunning difference in organization of connective tissue in *Labeotropheus* compared with *Tropheops*. Conith et al. (2018) found that the connective tissue (identified as the intermaxillary ligament) of *Labeotropheus*, which is high in collagen, invades the surrounding loose connective tissue and anchors to the epithelium potentially helping with the stiffness of the snout to improve foraging. The GO enrichment for DE genes between *O. nasuta* and *O. ventralis* revealed terms linked to cell fate and cell shape regulation, “peptidyl-proline modification” and “tendon development.” This suggests a difference in collagen/tendon development and cell adhesion and fate between the two Lake Tanganyika species, where the structure is not as unique as in *Labeotropheus* and shows an overall increase in the proportion of skin and other tissue, much greater than in *Labeotropheus* (Conith et al. 2018).

To understand if similar molecular mechanisms underly these morphologically similar (yet histologically different) phenotypes across both lakes, we looked at the intersection set of both comparisons and found many of DE genes, both upregulated and downregulated, that are associated with craniofacial development and involved in human dysmorphologies, many with midline facial defects including those that effect the nose in literature. Among the upregulated genes with related functions were *adprhl1* (De Pater et al. 2005), *angptl2* (Ehret et al. 2015), *colec12* (Zlotina et al. 2016), *cx43* (McLachlan et al. 2005), *foxa2* (Dines et al. 2019), *foxf1* (Kucharczyk et al. 2014), *galnt10* (Starkovich et al. 2016), *got1* (Tomkins et al. 1983), *lyve1* (Mitteldorf et al. 2018), *mdfic* (Kosho et al. 2008), *mid1* (Preiksaitiene et al. 2015; Hüning et al. 2013), *nudcd1* (Selenti et al. 2015), *pacs2* (Holder et al. 2012), *plxnb1* (Haldeman-Englert et al. 2009), *rac1* (Thomas et al. 2010; Reijnders et al. 2017), *rspo1* (Wieacker and Volleth 2007), *s100a10* (Sawyer et al. 2007), *slc25a18* (Chen et al. 2013), *slc6a6* (Kariminejad et al. 2015), *ugdh* (Alhamoudi et al. 2020), *vgll4* (Czeschik et al. 2014; Barrionuevo et al. 2014), and *vwa1* (Giannikou et al. 2012). Among the downregulated genes, we also found the following candidates to have such roles: *acsl1* (Yakut et al. 2015), *adgb* (Alazami et al. 2016), *arl13* (Brugmann et al. 2010), *ATP6v0c* (Mucha et al. 2019; Tinker et al. 2021), *bmp2* (Tan et al. 2017), *cntn3* (Ţuţulan-Cuniţǎ et al. 2012), *dusp22* (Hosono et al. 2020; Martinez-Glez et al. 2007), *fgf22* (Quigley et al. 2004), *gdpd3* (Dell’Edera et al. 2018), *grina* (Bonaglia et al. 2005), *hmx2* (Miller et al. 2009), *hoxa13* (Fryssira et al. 2011), *il23r* (Rivera-Pedroza et al. 2017), *ppp1r42* (Mordaunt et al. 2015), *prodh* (Guilmatre et al. 2010), *six2* (Hufnagel et al. 2016; Okello et al. 2017), *srsf3* (Pillai et al. 2019), *syt9* (Sofos et al. 2012), and *trpc2* (Sansone et al. 2014; Zhang et al. 2010). Interestingly, one of the downregulated genes, *pi15*, is known as an important molecular player in beak formation in birds (Nimmagadda et al. 2015). Even among the overlapping DE genes which showed opposing expression patterns between the two comparisons, we still found at least four genes to have been associated with craniofacial midline defects in other vertebrates, including *ccne2* (Jain et al. 2010), *plekha8* (Schulz et al. 2008), *rab1b* (Alwadei et al. 2016), *RBPJ* (Nakayama et al. 2014), and *tgfbr3* (Lopes et al. 2019). The most likely explanation for opposing expression of these genes can be the existence of bimodality in their expression pattern. Bimodality of gene expression is a mechanism contributing to phenotypic diversity (Ochab-Marcinek and Tabaka 2010), and it can be reflected by up- or downregulation of a gene during the same biological process. This regulatory bimodality can have various causes such as differential/opposing action of transcription factors (e.g., negative feedback loop), post-transcriptional factors (e.g., microRNA and circular RNA), and stochastic events. Interestingly, a highly conserved negative feedback loop in Notch signaling has already been shown to be mediated by opposing roles of RBPJ (Tanigaki and Honjo 2010). This indicates a potential bimodality of *rbpj* expression through a negative feedback loop in regulation of Notch signaling, which plays a crucial role in the formation of the midline structures including nasal structures (Tanigaki and Honjo 2010). Including developmental time series for expression profiling in future studies can help to identify whether the shared DEGs with opposing expressions also show bimodality in their expression.

These findings demonstrate that similar sets of genes are involved in midline patterning and growth across evolutionary distant vertebrates. Thus, functional studies investigating their specific role in divergent morphogenesis of snout structures in fish can provide valuable information about the conserved molecular mechanisms underlying the formation of facial soft tissues (Powder and Albertson 2016). Future studies with developmental time series, histological analyses, experimental crosses (particularly for species from Lake Malawi), and female *O. nasuta* are required to explore underlying mechanism of repeated evolution and to tease apart genes involved in snout development from those that only play a role in exaggeration of the snout.

Conducting GO enrichment analysis on the list of upregulated DE genes also revealed the involvement of several biological processes such as proline metabolism, “tendon development,” as well as Notch and Wnt signaling pathways (although Wnt signaling not significantly). Interestingly, defective proline and hydroxyproline metabolism has been associated with a range of skin and facial deformities including abnormal nose morphogenesis in humans (Kiratli and Satilmiş 1998; Kretz et al. 2011; Baumgartner et al. 2016; Zaki et al. 2016). Defective proline metabolism is known to severely affect collagen formation and extracellular matrix integrity and subsequently cell adhesion (Xinjie et al. 2001; Javitt et al. 2019; Velez et al. 2019; Karna et al. 2020; Noguchi et al. 2020). We found genes involved in “peptidyl-proline hydroxylation” enriched in the upregulated genes as well as “proline catabolic process” in the enrichment analysis of downregulated genes. In addition, it has been recently shown that the biosynthesis of proline is tightly regulated by TGFβ (Schwörer et al. 2020), a TF that also plays role in the early development of the flapped snout in cichlids (Conith et al. 2018). We also found components of this pathway (e.g., *tgfbr3*) to be differentially expressed, and both enriched pathways, Wnt and Notch, have conserved crosstalk with TGFβ signal in regulation of various molecular, cellular, and developmental events (Attisano and Labbé 2004; Chesnutt et al. 2004; Klüppel and Wrana 2005; Ahi 2016; Arnold et al. 2019). In addition, Wnt and Notch signaling pathways are known to play a pivotal role in craniofacial development and morphogenesis, including the formation of middle structures including nasal structures (Brugmann et al. 2007; Wang et al. 2011; Penton et al. 2012; Pakvasa et al. 2020; Singh et al. 2021).

The induction of TGFβ signaling is required for the establishment of cell–cell contacts in different tissues, whereas later induction of Notch signal stabilizes the TGFβ-mediated effects (Klüppel and Wrana 2005). In the context of the snout, it is possible that activation of TGFβ is required for early snout induction (Conith et al. 2018) and that continued snout growth is maintained via Notch signaling. This potential time-dependent crosstalk may be mediated through the downstream targets of Notch and TGFβ signals, because it is shown that both signals can regulate similar target genes (De Jong et al. 2004; Klüppel and Wrana 2005), including *foxa2*, a member of the FOX family of transcription factors (both signals suppress *foxa2* expression) (Liu et al. 2012; Kondratyeva et al. 2016). In our study, we found upregulation of *foxa2* in the flapped snout region, and interestingly, a recent study in human shows that a deletion in *Foxa2* can cause a variety of nasal deformities (Dines et al. 2019). Moreover, we found *rbpjb*, a major transcription factor mediating canonical Notch signal (Tanigaki et al. 2002), to be differentially expressed in the flapped snout of both species. In mice, *Rbpj* is shown to regulate a receptor of TGFβ signal (*Tgfbr1*), thus making a reciprocal positive regulatory loop between the two pathways (Valdez et al. 2012). We also found another receptor of TGFβ signal (*tgfbr3*) to show a similar expression pattern as *rbpjb* raising the possibility of the existence of such a reciprocal regulatory loop in flapped snout cichlids. In human, a deletion in *RBPJ* gene has been linked to abnormal thickening of the nose and lip (Nakayama et al. 2014). On the other hand, Bmp2 signal, which is regarded as another molecular cross point between TGFβ and Notch pathways (De Jong et al. 2004), mediates its signal through *Tgfbr3* (Hill et al. 2012). Previous studies in cichlids had proposed variations in Bmp expression as a molecular player in adaptive morphological divergence in different skeletal structures (Albertson et al. 2005; Gunter et al. 2013; Hulsey et al. 2016; Ahi et al. 2017). We found downregulation of *bmp2b* expression suggesting that a key regulator linking both pathways is affected in the flapped snout region. Furthermore, deletion of *Bmp2* in human has been reported to cause a range of nose and lip deformities (Tan et al. 2017). Taken together, these findings suggest complex interactions between Notch and TGFβ signals in the formation and possibly the maintenance of the flapped snout structure in cichlids.

Finally, we found several potential binding sites for TFs that may play a role in the formation of a flapped snout. The most represented TF binding sites belonged to members of FOX transcription factor family, for example, *foxd3*, *foxj1*, and *foxp1*, as well as a consensus binding site for the FOX family. In East African cichlids, both *foxd3* and *foxp1* were predicted to regulate a gene network involved in exaggerated fin elongation (Pashay Ahi and Sefc 2018; Ahi, Richter, et al. 2019). Additionally, *foxp1* was recently suggested as an upstream regulator of genes involved in the formation of the hypertrophic lip in another East African cichlid species (Lecaudey et al. 2021). None of the predicted FOX members (*foxd3*, *foxj1*, and *foxp1*) displayed consistent differential expression across both comparisons. It is, therefore, possible that the two other FOX members identified by RNA-seq and qPCR, *foxf1* and *foxa2*, are the key regulators of the entire list of DEGs, because they might bind to the predicted consensus FOX binding site. In addition, both *foxf1* and *foxa2* displayed consistently increased expression in the flapped snout in both comparisons and are also implicated in the nose morphogenesis in mammals (Kucharczyk et al. 2014; Dines et al. 2019). We have recently found *foxf1* among the differentially expressed genes in hypertrophied lips of an East African cichlid species as well (Lecaudey et al. 2021), suggesting a potential role of *foxf1* in soft tissues exaggeration in cichlids.

Among the other predicted TF binding site, we found overrepresentation of binding motif for *tcf12*, a transcription factor with known roles in development and morphogenesis of the frontal bone and cranial vault thickening in mammals (Sharma et al. 2013; Piard et al. 2015). We have previously identified *tcf12* as a potential key player in the formation of a nuchal hump in an East African cichlid (Lecaudey et al. 2019). In this study, we did not detect its differential expression in the snout. However, there might be other types of potential variations in these TFs (e.g., alternative splicing; Singh and Ahi 2022), which are not necessarily reflected in their overall expression differences, but still lead to changes in their regulatory effects. Interestingly, mutations causing missense, frame shift, and splicing changes are already reported for *tcf12*, which could lead to craniofacial deformities in humans (Sharma et al. 2013).

The only predicted TF with consistent expression difference in both comparisons was *ap4* (alias *tfap4*), that shows slight but significant reduced expression in the flapped snout. Transcription factor *ap4* encodes a member of the basic helix–loop–helix–zipper (bHLH-ZIP) family and can act as a transcriptional activator or repressor on a variety of downstream target genes mediating cell fate decisions (Wong et al. 2021). We also found both up- and downregulated genes among the predicted downstream targets of *ap4* (i.e., 93 genes contained ap4 binding site), which confirms its potential transcriptional activating or repressing roles. The exact role of *ap4* in craniofacial morphogenesis of soft tissues is unclear, and although deletions in a genomic region containing this gene cause facial dysmorphisms in humans such as prominent beaked nose and micrognathia (Gervasini et al. 2007), these phenotypes are mainly thought to be linked to mutations in a neighboring gene (*CBP* or *CREBBP*) in this region. Future functional studies are required to verify the potential role of *ap4* in formation and morphogenesis of craniofacial soft tissues in fish.

## Conclusions

The snout flap in *L. trewavasae* and *O. nasuta* is a striking and rare example of an exaggerated soft tissue trait that has evolved repeatedly in cichlid radiations of Lake Malawi and Lake Tanganyika, albeit for different functions. Comparing the transcriptional landscape of the snout flap tissue of these two species with the snout of close relatives that do not develop such a structure, we identified 201 genes that were repeatedly recruited to give rise to the snout flap phenotype even after >9.0 Ma of divergence. Our study provides support for a change in proline hydroxylation, a mechanism also linked to human facial deformations, to be a mechanism for metabolic changes involved in the formation of the snout flap in fish. We found complex interactions between the TGFβ, regulating the biosynthesis of proline, and Notch signalling, which are known players in craniofacial development and morphogenesis, in the formation and maintenance of the snout flap. We identified transcription factors belonging to the FOX family (especially *foxf1* and *foxa2*) which are both linked to nose morphogenesis in mammals and *ap4*, a transcription factor that is transcriptionally repressed in species with a snout flap, but with a previously unknown role in craniofacial soft tissue formation. Our study emphasizes that studying the genes involved in fish snout morphogenesis can shed light on the conserved molecular mechansisms crucial for the development of soft facial tissues. In the future, it would be important to build on these findings and confirm the reuse of these genes and pathways across more distant teleost groups.

## Materials and Methods

### Fish Rearing and Tissue Sampling

Five captive-bred males of each *O. nasuta* and *O. ventralis* and five captive-bred females of *L. trewavasae* and *T. tropheops* were raised and kept in a large tank (approximately 450 l) containing multiple stony shelters to decrease competition stress. All specimens were at the young adult stage and have been fed with the same diet, tropical multi-ingredient flakes suitable for omnivorous cichlids. The two species in each comparison were sampled at the same time when the protrusion of the flapped snout had already appeared (fig. 1). To perform the dissections, we used a solution with 0.3 g MS222 per 1 l water to euthanize the fish, and similar snout regions, an area above the upper lip encompassing the nostrils which includes epidermis, dermis, and the underlying soft connective tissues, were sampled for each fish (fig. 1). The sampled snout tissues for each individual were placed into separate tubes containing RNAlater (Qiagen) and stored at −20 °C. The sacrificing of fish followed the guidelines of the Federal Ministry of Science, Research and Economy of Austria according to the regulations of the BMWFW.

### RNA Extraction and cDNA Synthesis

Total RNA was extracted from 20 dissected snout tissue samples (five biological replicates per species) following the TRIzol method (Thermo Fischer Scientific). Each dissected sample included epidermis, dermis, and the underlying fibrous/connective tissues of the specified nose regions (fig. 1). Tissue samples were placed into tubes containing 1 ml of TRIzol with a ceramic bead (1.4 mm) and homogenized using a FastPrep-24 Instrument (MP Biomedicals, CA, USA). RNA extraction followed the protocol of TRIzol RNA extraction from Thermo Fischer Scientific. A DNA removal step with DNase followed the extraction (Invitrogen). The total RNAs were dissolved in 50 *µ*l nuclease-free water, and their concentrations were quantified through a Nanophotometer (IMPLEN GmbH, Munich, Germany). We measured the quality of RNAs with the R6K ScreenTape System using an Agilent 2200 TapeStation (Agilent Technologies) and RNA integrity numbers (RIN) above 7 were aimed at for all samples. To synthesize cDNA for qPCR analysis, we used 500 ng of the total RNA per sample and followed the manufacturer’s protocol of the High Capacity cDNA Reverse Transcription kit (Applied Biosystems), and the resulted cDNAs were diluted 1:4 to be used for the qPCR reaction.

### RNA-seq Library Preparation and Gene Expression Quantification

To attain transcriptome data of the snout tissues, we conducted RNA-seq library preparation with 1000 ng of total RNA per tissue sample as input and following the protocol of the Standard TruSeq Stranded mRNA Sample Prep Kit (Illumina) with indexing adapters. The library qualities were assessed using D1000 ScreenTape and reagents (Agilent) on a TapeStation 2200 machine (Agilent). In order to reach an optimal quantity recommended for sequencing, we diluted the libraries and pooled them with equal molar concentration for each library. The RNA-sequencing was conducted in the NGS Facility at Vienna Biocenter Core Facilities (VBCF, Austria) on an Illumina HiSeq2500 and generated between 6.7 and 15.8 million paired-end reads with 125 bp length per sample (supplementary table S1, Supplementary Material online). Raw reads were demultiplexed based on unique barcodes by the same facility. The quality of the reads was assessed with FastQC (v0.11.8) (Andrews 2012), and reads were filtered for a quality >28 and a minimum length of 70 bp with Trimmomatic (v0.3.9) (Bolger et al. 2014). Reads were aligned to the *Oreochromis niloticus* reference genome (Conte et al. 2017) of the University of Maryland using RNAstar (v2.7.3.a) (Dobin et al. 2013). To check the mapping statistics, we used samtools idxstats (v1.9) (Danecek et al. 2021) (supplementary table S1, Supplementary Material online) and further merged the single files for species and lake with picard (v2.21.7) (Picard Toolkit. 2019. Broad Institute, GitHub Repository, https://broadinstitute.github.io/picard/). We used StringTie (v.2.0.6) (Pertea et al. 2015) to assemble the alignments into potential transcripts without a reference. This step was conducted separately for single files (per biological replicate) and the merged files (per species and per lake). The single files per biological replicate were further merged into species. This process of repeated merging steps was implemented to reduce the probability of false positives. To assess the accuracy of the mapping, we used gffcompare (v0.11.2) (Pertea and Pertea 2020) to compare our annotations with the reference annotation. Subsequently, we filtered for monoexonic transcripts that were not contained in our reference and the transcripts assigned the class code “possible polymerase run-on” by gffcompare. As the maximum intron length of the *O. niloticus* reference is 200,000 bp, we also filtered for that in the produced annotation. The expression estimates for each transcript were based on these annotations and generated with StringTie (v.2.0.6) with no multimapping allowed. The final raw count matrices were produced from the expression estimates with a Perl script from the griffith lab (https://github.com/griffithlab/rnaseq_tutorial/blob/master/scripts/stringtie_expression_matrix.pl), and the code used in this analysis is available at this github repository (https://github.com/annaduenser/snout_flap_RNAseq).

Differential expression analysis was conducted using DESeq2 (Love et al. 2014) in R (R Core Team 2017) running comparisons for each lake separately. DESeq2 estimates variance–mean dependence based on a model using negative binomial distribution using the raw counts (Love et al. 2014). A FDR of *P* < 0.05 was chosen as the cutoff for the adjusted *P* value to determine differentially expressed genes.

For the downstream analysis, an enrichment step for GO terms of biological processes was conducted in R using topGO (v2.48.0) (Alexa and Rahnenfuhrer 2019) with the method *weight* and using Fisher's exact tests for the enrichment analysis, while GO terms for *O. niloticus* were acquired via the biomaRt package (v2.46.1) (Durinck et al. 2005, 2009) from the Ensemble database. To further group functionally similar GO terms, we also used REVIGO (Supek et al. 2011) using simRel scores (Schlicker et al. 2006). To predict the potential upstream regulators of DE genes, we conducted motif overrepresentation analysis on 4 kb upstream sequences (promoter and 5′-UTR) of these genes using the annotated genome of the Nile tilapia (Zerbino et al. 2018) and MEME tool (Bailey et al. 2009). The motifs that were present in the promoters of at least one-fifth of the total 201 DEGs were compared with position weight matrices (PWMs) in the TRANSFAC database (Matys et al. 2003) via STAMP (Mahony and Benos 2007) in order to identify matching TF binding sites. In addition, we investigated the functional interactions between the products of DE genes through STRING v10 (http://string-db.org/), a knowledge-based interaction prediction tool, and zebrafish databases for protein interactomes (Szklarczyk et al. 2017).

### Primer Design and qPCR

We designed the qPCR primers on conserved regions of the selected candidate genes by aligning their assembled sequences to their already available homologous mRNA sequences from *O. ventralis* (Böhne et al. 2014), *Metriaclima zebra*, *Pundamilia nyererei*, *Neolamprologus brichardi*, and *Astatotilapia burtoni* (Brawand et al. 2014), as well as *O. niloticus*. After aligning the conserved sequence regions across the abovementioned East African cichlids, we identified the exon junctions (using CLC Genomic Workbench, CLC Bio, Denmark, and annotated genome of *A. burtoni* in the Ensembl database, http://www.ensembl.org). The primer designing steps were conducted as described previously (Pashay Ahi and Sefc 2018; Ahi, Richter, et al. 2019) using Primer Express 3.0 (Applied Biosystems, CA, USA) (supplementary table S5, Supplementary Material online). The qPCR was performed based on the protocol provided by Maxima SYBR Green/ROX qPCR Master Mix (2X) (Thermo Fisher Scientific, Germany) following the guidelines for optimal experimental setup for each qPCR run (Hellemans et al. 2007). The qPCR program was set for 2 min at 50 °C, 10 min at 95 °C, 40 cycles of 15 s at 95 °C, and 1 min at 60 °C, followed by an additional step of dissociation at 60–95 °C. The primer efficiency (*E*-values) for each gene was calculated through standard curves generated by serial dilutions of pooled cDNA samples. The standard curves were run in triplicates and calculated using the following formula: *E* = 10(−1/slope) (supplementary table S5, Supplementary Material online).

In order to select stably expressed candidate reference genes, we filtered for genes with a low log_2_ fold change and subsequently ranked the remaining genes according to low coefficient of variation. The top six most stable genes shared across the transcriptome comparisons were selected as candidate reference genes (supplementary table S4, Supplementary Material online). After qPCR expression analysis of the six genes across all samples, we ranked them based on their expression stability by three different algorithms: BestKeeper (Pfaffl et al. 2004), NormFinder (Andersen et al. 2004), and geNorm (Vandesompele et al. 2002). We used the Cq values of the top most stable reference genes to normalize Cq values of target genes in each sample (ΔCq target = Cq target − Cq reference). The relative expression levels (RQ) were calculated by 2^−ΔΔCq^ method (Pfaffl 2001), and the log-transformed RQ values were used for independent samples *t*-tests to calculate the statistical differences.

## Supplementary Material

evad045_Supplementary_Data

## Data Availability

The data underlying this article are available in the Sequencing Read Archive (SRA) of NCBI at https://www.ncbi.nlm.nih.gov/ and can be accessed with PRJNA770252.
