# Supplementary material for: Conserved Molecular Players Involved in Human Nose Morphogenesis Underlie Evolution of the Exaggerated Snout Phenotype in Cichlids
Source: Genome Biol Evol. 2023 Mar 17;15(4):evad045. doi: 10.1093/gbe/evad045 (PMC10078796; doi:10.1093/gbe/evad045)
Supplement: evad045_Supplementary_Data [file evad045_supplementary_data.zip › Supplementary_Analysis.docx]

**Supplementary Analysis**

To check whether the difference in the number of differentially expressed genes between the two within lake comparisons was caused by the use of differing genera for the Lake Malawi comparison, we constructed a divergence tree based on transcriptome data (Fig. S1). Looking at the branch length representing the relative number of changes per site, a difference in number of differentially expressed genes can probably not be explained by the use of differing genera. Additionally, no difference can be seen between the tree based on the subset of shared genes (Fig. S1 B) versus the tree based on the subset of genes that are differentially expressed within each lake and not shared between the lakes (Fig. S1 A).

**Fig. S1.** **Divergence tree based on one hundred concatenated genes.** For (A) non overlapping genes within the Lake comparisons and (B) overlapping genes between the Lake comparisons. Node labels include bootstrap support followed by the branch length (relative number of changes per site (total number of sites (A): 215414 sites considered, (B): 220690 sites considered))

**Supplementary Methods**

A fasta file containing the nucleotide sequences for each sample using a majority rule algorithm from the mapped bam files was created (angsd) (Korneliussen et al. 2014). Using gffread (Pertea & Pertea 2020), sequences for each transcript were extracted using the generated annotation file (used for differential gene expression analysis). To reduce computational time a subset of one hundred genes each, which were differentially expressed in each of the lakes and which were differentially expressed in both of the lakes, was used. For one transcript per gene a best model was calculated and used for partitioned analysis. With the concatenated sequence file of all transcripts and the partitions a maximum likelihood (LM) tree was generated and supported with one hundred bootstrap samples (raxml-ng, raxmlGUI) (Kozlov et al. 2019, Edler et al. 2020).

**Supplementary References**

Edler D, Klein J, Antonelli A, Silvestro D. 2020. raxmlGUI 2.0: A graphical interface and toolkit for phylogenetic analyses using RAxML. Methods Ecol. Evol. 12:373-377 doi: http://dx.doi.org/10.1111/2041-210X.13512.

Korneliussen TS, Albrechtsen A, Nielsen R. 2014. ANGSD: Analysis of Next Generation Sequencing Data. BMC Bioinformatics. 15:1-13. https://doi.org/10.1186/s12859-014-0356-4.

Kozlov AM, Darriba D, Flouri T, Morel B, Stamatakis A. 2019. RAxML-NG: A fast, scalable, and user-friendly tool for maximum likelihood phylogenetic inference. Bioinformatics. 35:4453-4455. doi:10.1093/bioinformatics/btz305.

Pertea G, Pertea M. 2020. GFF Utilities: GffRead and GffCompare. F1000Research. 9:1-19. doi: 10.12688/f1000research.23297.2.
