# Supplementary figures and images for: Conserved Molecular Players Involved in Human Nose Morphogenesis Underlie Evolution of the Exaggerated Snout Phenotype in Cichlids
Source: Genome Biol Evol. 2023 Mar 17;15(4):evad045. doi: 10.1093/gbe/evad045 (PMC10078796; doi:10.1093/gbe/evad045)

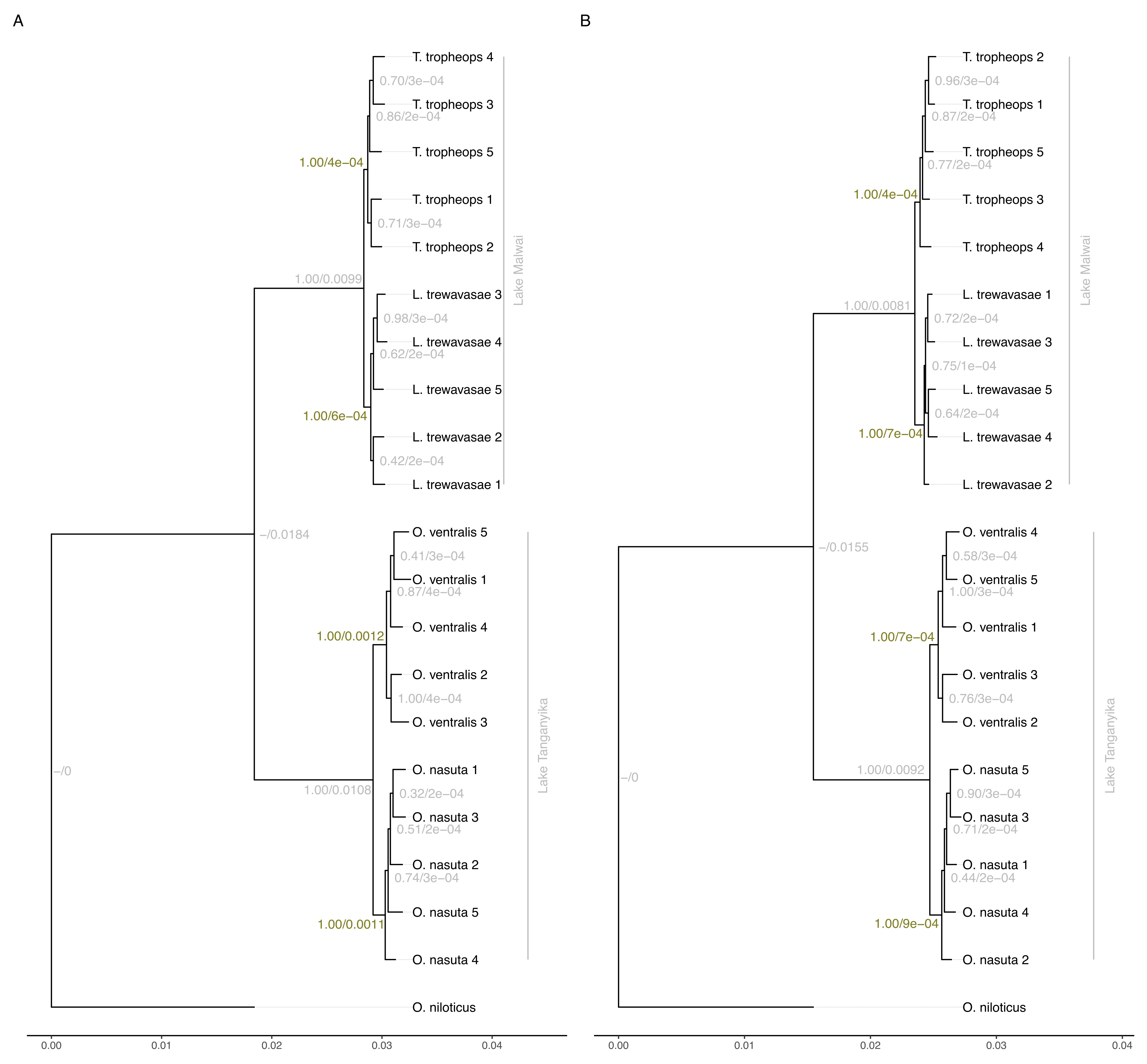

Supplement: evad045_Supplementary_Data [file evad045_supplementary_data.zip › Fig_S1.jpg]
